# Supplementary material for: Assessing feasibility and maternal acceptability of a biomechanically-optimized supine birth position: A pilot study
Source: PLoS One. 2021 Sep 10;16(9):e0257285. doi: 10.1371/journal.pone.0257285 (PMC8432866; doi:10.1371/journal.pone.0257285)
Supplement: S1 Protocol — (PDF) [file pone.0257285.s002.pdf]

A qualitative study to assess the feasibility and acceptability of an optimized birthing position.

Study Type:	Other Clinical Trial according to ClinO, Chapter 4
Risk Categorisation:	Category A
Study Registration:	1. WHO International Clinical Trials Registry Platform (ICTRP; http://www.who.int/ictrp/en/).<br>2. FOPH portal SNCTP (Swiss National Clinical Trial Portal).
Project Leader:	Dr David Desseauve – Médecin cadre – MD, PhD, PD-MER<br>Département femme-mère-enfant, CHUV<br>Avenue Pierre-Decker 2, 1011 Lausanne<br>E-Mail: David.desseauve@chuv.ch<br>Phone: 079.556.82.59
Co-investigator:	Lisa Bouille<br>E-Mail: lisa.bouille@unil.ch
Investigated Intervention:	Optimized birthing position
Protocol ID:	2019-00872
Version and Date:	Version 3 (dated 18/07/2019)

PROTOCOL SIGNATURE FORM

Study Title	A qualitative study to assess the feasibility and acceptability of an optimized birthing position
Study ID	2019-00872

The Project Leader and the Obstetric-Gynaecology department of the CHUV has approved the protocol version 2 (dated 18/07/2019) and confirm hereby to conduct the study according to the protocol, current version of the World Medical Association Declaration of Helsinki, and ICH-GCP guidelines as well as the local legally applicable requirements.

Project Leader:

Name: David Desseauve

Date: ____________________________ Signature: _______________________________

Co-investigator:

Name: Lisa Bouille

Date: ____________________________ Signature: _______________________________

TABLE OF CONTENTS

TABLE OF CONTENTS 4

GLOSSARY OF ABBREVATIONS 5

1 BACKGROUND AND RATIONALE 6

2 STUDY OBJECTIVES AND DESIGN 6

2.1 Hypothesis and primary objective 6

2.2 Primary and secondary endpoints 6

2.3 Study design 7

2.4 Study intervention 7

3 STUDY POPULATION AND STUDY PROCEDURES 7

3.1 Inclusion and exclusion criteria, justification of study population 7

3.2 Recruitment, screening and informed consent procedure 7

3.3 Study procedures 8

3.4 Withdrawal and discontinuation 8

4 STATISTICS AND METHODOLOGY 8

4.1. Statistical analysis plan and sample size calculation 8

4.2. Handling of missing data and drop-outs 8

5 REGULATORY ASPECTS AND SAFETY 9

5.1 Local regulations / Declaration of Helsinki 9

5.2 (Serious) Adverse Events 9

5.3 (Periodic) safety reporting 10

5.4 Radiation 10

5.5 Pregnancy 10

5.6 Amendments 10

5.7 (Premature) termination of study 10

5.8 Insurance 11

6 FURTHER ASPECTS 11

6.1 Overall ethical considerations and risk-benefit assessment 11

7 QUALITY CONTROL AND DATA PROTECTION 11

7.1 Quality measures 11

7.2 Data recording and source data 11

7.3 Confidentiality and coding 12

7.4 Retention and destruction of study data and biological material 12

8 MONITORING AND REGISTRATION 12

9. FUNDING / PUBLICATION / DECLARATION OF INTEREST 12

10 REFERENCES 12

GLOSSARY OF ABBREVATIONS

AE	*Adverse Event*
ASR/DSUR	*Annual Safety Report / Development Safety Report*
BASEC	*Business Administration System for Ethical Committees*
CRF	*Case Report Form*
CS	*Caesarean Section*
CTCAE	*Common Terminology Criteria for Adverse Events*
FADP	*Federal Act on Data Protection*
FOPH	*Federal Office of Public Health*
GCP	*Good Clinical Practice*
HRA	*Human Research Act*
ICH	*International Conference on Harmonisation*
ClinO	*Ordinance on Clinical Trials in Human Research*
OPTIMAC	*OPTimisation des Mécanismes de l'aCcouchement*
SAE	*Serious Adverse Event*

1 BACKGROUND AND RATIONALE

Recent data brings out that the CS rate in Switzerland is quite high, reaching 33.4 % (8). Though, the reasons to this increasing amount of CS are not fully known. Therefore, it is absolutely necessary to expand the existing knowledge in different fields, including the biomechanics of childbirth. Numerous CS are performed in response to an obstructed labour, especially when the foetus does not engage in the pelvis near full dilatation. In order to manage such situations, midwifes currently position parturients in pragmatic postures. Although not verified by data, this care management suggests that it is possible to impact the position of the bone segments at stake for vaginal birth including pelvis and lumbar spine and to promote the descent of the foetus through the pelvic inlet plane. In his PhD thesis about vaginal birth biomechanics, Desseauve et al investigated this area and found out that an optimized position similar to the squatting position (hyperflexion of the thighs and loss of the lumbar lordosis) could be close to the perfect delivery position in terms of ability for the foetus to go through the pelvic inlet plane (9). Although these findings are encouraging, it is yet to be confirmed in clinical practice, particularly when a dystocia occurs (10). Prior to doing that, it is though necessary to validate the optimized posture in terms of acceptability in a qualitative clinical study. In this study, fifteen to twenty parturients who respond to the inclusion criteria and whose foetus does not engage in the pelvis near full dilation will be asked to adopt the optimized position for a twenty minutes period. The investigator will then consign information reflecting the progress of the labour on a data sheet. As during labour midwifes already position parturients in various postures, some of which very similar to the suggested optimized position, it is sensible to place the study in the risk A category according to ClinO, Art. 61.

2 STUDY OBJECTIVES AND DESIGN

2.1 Hypothesis and primary objective

The hypothesis is that the optimized birthing position, as described in the first chapter of this protocol, is an acceptable and feasible position. This means that the included parturients will be able to maintain the optimized birthing position for a certain amount of time without giving up or feeling uncomfortable achieving it. The primary objective of the study is thus to determine, by clinically testing the optimized position on a reduced sample of parturients, if it is well accepted by them with a satisfaction scale.

2.2 Primary and secondary endpoints

In order to test the hypothesis, the primary endpoint will be whether or not the participants manage to maintain the optimized birthing position for the twenty minutes duration of the study, and if they do, how well they manage it. Some variables like age, parity or history of difficult delivery will be described for each participant. In complement to the primary endpoint, the number of parturients who will refuse to participate to the study will give additional insight into the acceptability of the optimized birthing position. If the reason of their refusal is mentioned, it will then be used to give an additional insight into the acceptability of the optimized birthing position.

The secondary endpoint variables will include:

- General information
- Risk factors of non-engagement of the foetus at full dilation
- Foetal parameters
- Delivery outcome

- Adverse events
- Presence of symphyseal separation

However, this study was not design to obtain significant results regarding the secondary endpoint variables.

2.3 Study design

This study is a Swiss monocentric non-randomised qualitative study.

2.4 Study intervention

The intervention will consist in positioning the parturient in an optimized position. It means the parturient will be asked to adopt a supine position with a hyperflexion of the thighs and a flat back, and to maintain the position for twenty minutes. This period of time represents approximately twenty uterine contractions and will give the opportunity to appreciate the effectiveness of the measure without being to constraining for the participants. Most of the time, the patients indeed have epidural anaesthesia and it is though not recommended to make them adopt a position for a prolonged amount of time, in order to avoid any risk of malposition or nerve compression. Help from the investigator or the midwife in charge will be provided if needed or requested. The labour will continue during the twenty minutes period, as obstetrical labour once started can’t be interrupted.

3 STUDY POPULATION AND STUDY PROCEDURES

3.1 Inclusion and exclusion criteria, justification of study population

The study population will be constituted of twenty parturients with interrupted labour due to the non-engagement of the foetus in the pelvis at full dilation. Only the parturients responding to the inclusion criteria will participate to the study.

Study inclusion criteria:

- Informed consent as documented by signature
- Parturient in situation of mechanical dystocia (non engagement at full dilation)

Study exclusion criteria:

- Inability or medical contraindications to undergo the investigated intervention (e.g. orthopaedic injury or disease preventing the parturient from adopting the optimised position)
- Clinically significant concomitant diseases
- Incapacity of judgment
- Inability to follow the procedures of the study due to language problems, psychological disorders, dementia, etc.
- Foetus cardiac rhythm disorder

3.2 Recruitment, screening and informed consent procedure

The recruitment of the participants will begin by information during the antenatal consultation in the CHUV polyclinic of gynaecology and obstetrics. The patients will receive the participant information sheet and oral information from the physician in charge or from the co-investigator accompanied by a physician. Il they are willing to participate to the study, they will have to sign

the consent form directly at the end of the consultation.

The physician or the co-investigator will explain to each patient the nature of the study, its purpose, the procedures involved, the expected duration, the potential risks and benefits and any discomfort it may entail. Each participant will be informed that the participation in the study is voluntary and that she may withdraw from the study at any time and that withdrawal of consent will not affect her subsequent medical assistance and treatment, or the medical assistance of the foetus.

The participant will be informed that authorised individuals other than their treating physician, including the project leader (Dr Desseauve), her research assistant (Anna Fernandez) and the co-investigator (Lisa Bouille), may examine her medical records.

All participants for the study will be provided a participant information sheet and a consent form describing the study and providing sufficient information for participant to make an informed decision about their participation in the study. A frame of twenty minutes will be given to the parturient in order to decide whether or not she will participate to the study.

The consent form will be signed and dated by the participant and a physician. All the consent forms will be signed during the consultation preceding the admission in the labour ward. A copy of the signed informed consent will be given to each study participant. The consent form will be retained as part of the study records. The informed consent process will be documented in the patient file and any discrepancy to the process described in the protocol will be explained.

At the admission in the labour ward, an oral confirmation of the informed consent will be asked in presence of the co-investigator and the midwife to make sure the parturient still wants to participate to the study.

3.3 Study procedures

The planned overall study duration will be of approximately one hour per participant, and will include a recruitment period of thirty minutes and study duration of twenty minutes.

3.4 Withdrawal and discontinuation

A participant will be withdrawn from the study in case of withdrawal of informed consent, disease progression, occurrence of foetus cardiac rhythm disorder or labour progression. When a withdrawal occurs, data available will be collected, anonymised, used and archived using the same method as completed data from another participant.

4 STATISTICS AND METHODOLOGY

4.1. Statistical analysis plan and sample size calculation

In this observational pilot study, no sample size was defined.

4.2. Handling of missing data and drop-outs

Dropouts will be replaced by recruitment of subsequent new subjects. The data of the participants dropping out from the study before the end of the twenty minutes period will be handled and archived the same as the remaining data. The number of drop-outs will indeed contribute to assess the acceptability of the position.

5 REGULATORY ASPECTS AND SAFETY

5.1 Local regulations / Declaration of Helsinki

This study is conducted in compliance with the protocol, the current version of the Declaration of Helsinki, the ICH-GCP, the HRA as well as other locally relevant legal and regulatory requirements.

5.2 (Serious) Adverse Events

An Adverse Event (AE) is any untoward medical occurrence in a patient or a clinical investigation subject which does not necessarily have a causal relationship with the trial procedure. An AE can therefore be any unfavourable or unintended finding, symptom, or disease temporally associated with a trial procedure, whether or not related to it.

A Serious Adverse Event (SAE) (ClinO, Art. 63) is any untoward medical occurrence that

- Results in death or is life-threatening,
- Requires in-patient hospitalisation or prolongation of existing hospitalisation,
- Results in persistent or significant disability or incapacity, or
- Causes a congenital anomaly or birth defect

The Project Leader makes a causality assessment of the event to the trial intervention, (see table below based on the terms given in ICH E2A guidelines). Any event assessed as possibly, probably or definitely related is classified as related to the trial intervention.

Relationship	Description
Definitely	Temporal relationship<br>Improvement after dechallenge*<br>Recurrence after rechallenge<br>(or other proof of drug cause)
Probably	Temporal relationship<br>Improvement after dechallenge<br>No other cause evident
Possibly	Temporal relationship<br>Other cause possible
Unlikely	Any assessable reaction that does not fulfil the above conditions
Not related	Causal relationship can be ruled out
*Improvement after dechallenge only taken into consideration, if applicable to reaction	

The Project Leader makes a severity assessment of the event as mild, moderate or severe. Mild means the complication is tolerable, moderate means it interferes with daily activities and severe means it renders daily activities impossible.

Reporting of SAEs (see ClinO, Art. 63)

All SAEs are documented and reported immediately (within a maximum of 24 hours) to the Project Leader of the study.

If it cannot be excluded that the SAE occurring in Switzerland is attributable to the intervention under investigation, the Project Leader reports it to the Ethics Committee via BASEC within 15 days.

Follow up of (Serious) Adverse Events

In case of reported ongoing (S)EAs, the participants will stay in the hospital or will be under the care of an obstetrical physician in the CHUV polyclinic of gynaecology and obstetrics until resolution or stabilisation of the (S)EAs.

5.3 (Periodic) safety reporting

At the end of the study a safety report (ASR/DSUR) will be submitted once a year (according to the duration of the study) to the local Ethics Committee by the Project Leader (ClinO, Art. 43 Abs).

5.4 Radiation

Not applicable

5.5 Pregnancy

Reporting of pregnancies is not relevant in this study as all participants are pregnant women.

5.6 Amendments

Substantial changes to the study setup and study organization, the protocol and relevant study documents are submitted to the Ethics Committee for approval before implementation. Under emergency circumstances, deviations from the protocol to protect the rights, safety and well being of human subjects may proceed without prior approval of the Ethics Committee. Such deviations shall be documented and reported to the Ethics Committee as soon as possible.

Substantial amendments are changes that affect the safety, health, rights and obligations of participants, changes in the protocol that affect study objective(s) or central research topic, changes of study site(s) or of study Project Leader (ClinO, Art. 29).

5.7 (Premature) termination of study

The Project Leader may terminate the study prematurely according to certain circumstances, e.g.

- Ethical concerns,
- Insufficient participant recruitment,
- When the safety of the participants is doubtful or at risk (e.g. when the benefit-risk assessment is no longer positive), in particular if sensitive or motor nervous lesions occur.
- Alterations in accepted clinical practice that make the continuation of the study unwise, or
- Early evidence of harm or benefit of the experimental intervention

Upon regular study termination, the Ethics Committee is notified via BASEC within 90 days (ClinO, Art. 38).

Upon premature study termination or study interruption, the Ethics Committee is notified via BASEC within 15 days (ClinO, Art. 38).

All health-related data are anonymised upon end of data analysis.

5.8 Insurance

In the event of study-related damage or injuries, the liability of the Obstetric-Gynaecology department of the CHUV institution provides compensation.

6 FURTHER ASPECTS

6.1 Overall ethical considerations and risk-benefit assessment

Participants will receive complete written information and their written consent will be required to enable them to participate to the study. Given that this is a pilot study to investigate the acceptability of the optimized position, the data gathered is intending for quite a specific purpose. This study is though the first step toward the acceptability of an optimized childbirth position that we could use in case of an obstructed labor. A more formal definition of the optimized position is in progress in the project OPTIMAC, including a biomechanics laboratory (Swiss motion Lab) and the EPFL. This optimized position would be close to the position described in this study. That is the reason why we want to test its acceptability.

If the optimized position proves to be acceptable and demonstrate its efficiency to solve obstructed labor without medical intervention, the present study will path the way to a better knowledge about obstetrical biomechanics and to the decrease of the CS rate when a situation of dystocia occurs.

This study will not impact the management of dystocia and will thus not affect the health care of the participants and the foetus.

7 QUALITY CONTROL AND DATA PROTECTION

7.1 Quality measures

Double data entry will be performed for the primary outcomes. For the rest of the data, a random 5% will be double-checked. For quality assurance the Project Leader or the Ethics Committee may visit the research sites. Direct access to the source data and all study related files are granted on such occasions. All involved parties keep the participants data strictly confidential.

7.2 Data recording and source data

Each patient will receive a specific and unique number. A separate document (key) will be created in parallel allowing correlation of the data with the respective patients. All data necessary to the study will firstly be gathered on paper Case Report Forms (CRF), and will be coded before entry to our database by the co-investigator (Lisa Bouille). The key document will thereafter be entrusted to the project leader and will be stored separately from the other documents. The coded database will be accessible to the project leader and the co-investigator. During the analysis of the data, the co-investigator will take appropriate measures to maintain the confidentiality of the patients and restrict access to all non-authorized persons. The coded database will be password protected and stored on a secure CHUV server. The coded information will be handled with respect to the instructions present in this protocol, and according to relevant data protection regulations.

7.3 Confidentiality and coding

Trial and participant data will be handled with uttermost discretion and is only accessible to authorized personnel who require the data to fulfill their duties within the scope of the study. On the CRFs and other study specific documents, participants are only identified by a unique participant number.

The coded data will be transferred to a protected Excel document stored on a secure server of the CHUV. To ensure that data is secure, we will thereafter regularly draft dated PDF copies, which will then be again signed and dated by hand by the project leader. We will thus be able to monitor any modifications performed. The printed copies of the Excel spreadsheets and CRF will be safeguarded in a locked cabinet in Dr Desseauve's office. The Project Leader and the co-investigator alone will have access to this cabinet and will have access to the project plan, dataset, statistical codes, etc. during and after the research project (publication, dissemination).

Biological material in this study is not used. Only non-genetic data are used.

7.4 Retention and destruction of study data and biological material

All study data will be archived in the department of Gynaecology and Obstetrics of the CHUV institution for a minimum of 10 years after study termination or premature termination of the study. Data will be anonymised before the archiving and the coding document (the coding key) will thus be destroyed at the end of the study.

8 MONITORING AND REGISTRATION

In this pilot study, the investigators will be attentive to the reporting of eventual adverse event during labor and in the following days. Pr Yvan Vial, doctor in the department of Gynaecology and Obstetrics of the CHUV, will perform the monitoring of the study. A peer protocol reviewed by the research team and Pr Yvan Vial will be planned when the first fives inclusions will be done. All the data will be accessible by the Pr Yvan Vial and on demand by the CHUV institution.

Registration in French in the Swiss National Clinical trial Portal (SNCTP via BASEC) is planned. In addition, the study will be registered in the WHO International Clinical Trials Registry Platform (ICTRP; http://www.who.int/ictrp/en/).

9. FUNDING / PUBLICATION / DECLARATION OF INTEREST

There aren't any publication policy or possible conflict of interests in relation to the study. All the data will be anonymised at the end of data analysis. Though this is unlikely to happen, the Leenards foundation will bear the cost of eventual fees the study would cause.

10 REFERENCES

1. Common Terminology Criteria for Adverse Events (CTCAE) https://www.eortc.be/services/doc/ctc/CTCAE_4.03_2010-06-14_QuickReference_5x7.pdf
2. Declaration of Helsinki https://www.wma.net/policies-post/wma-declaration-of-helsinki-ethical-principles-for-medical-research-involving-human-subjects/
3. Federal Act on Data Protection (FADP) https://www.admin.ch/opc/en/classified-compilation/19920153/index.html

4. Human Research Act (HRA) https://www.admin.ch/opc/de/classified-compilation/20061313/index.html
5. International Conference on Harmonization (ICH) E6(R2) Guideline for Good Clinical Practice http://www.ich.org/fileadmin/Public_Web_Site/ICH_Products/Guidelines/Efficacy/E6/E6_R2__Step_4_2016_1109.pdf
6. International Conference on Harmonization (ICH) E2A Clinical Safety Data Management: Definitions and Standards for Expedited Reporting http://www.ema.europa.eu/docs/en_GB/document_library/Scientific_guideline/2009/09/WC500002749.pdf
7. Ordinance on Clinical Trials in Human Research (ClinO) https://www.admin.ch/opc/de/classified-compilation/20121176/index.html
8. OFS – Statistique médicale des hôpitaux (MS) https://www.bfs.admin.ch/bfs/fr/home/statistiques/catalogues-banques-donnees/cartes.assetdetail.4262551.html
9. Desseauve D. Toward a better understanding of the role of birthing position in obstetrical mecanics : Biomechanical analysis of segmental postures. PhD Thesis, Poitiers (FR) University, 2018.
10. Desseauve D, Fradet L, Lacouture P, Pierre F. Position for labor and birth : State of knowledge and biomechanical perspectives. Eur J Obstet Gynecol Reprod Biol. janv 2017;208:46-54.
11. Gupta JK, Sood A, Hofmeyr GJ, Vogel JP. Position in the second stage of labour for women without epidural anaesthesia. *Cochrane Database of Systematic Reviews* 2017, Issue 5. Art. No.: CD002006
12. De Jong PR, Johanson RB, Baxen P, *et al.* Randomised trial comparing the upright and supine positions for the second stage of labour. *Br J Obstet Gynaecol* 1997;104;567-71
13. A. De Jonge & A. L. M. Lagro-Janssen. Birthing positions. A qualitative study into the views of women about various birthing positions. *Journal of Psychosomatic Obstetrics & Gynecology* 2004, 25:1, 47-55
14. A. De Jonge, T. A. M. Teunissen & A. L. M. Lagro-Janssen. Supine position compared to other positions during the second stage of labor : a meta-analytic review, *Journal of Psychosomatic Obstetrics & Gynecology*, 25:1, 35-45
